# Summary of clinical and laboratory data of study subjects with and without DCE-MRI plaque measurements in the AIM-HIGH clinical trial

**DOI:** 10.1016/j.dib.2015.12.030

**Published:** 2016-01-02

**Authors:** Kevin D. O’Brien, Daniel S. Hippe, Huijun Chen, Moni B. Neradilek, Jeffrey L. Probstfield, Suzanne Peck, Daniel A. Isquith, Gador Canton, Chun Yuan, Nayak L. Polissar, Xue-Qiao Zhao, William S. Kerwin

**Affiliations:** a Division of Cardiology, Department of Medicine, University of Washington, Seattle, WA, USA; b Department of Radiology, University of Washington, Seattle, WA, USA; c Center for Biomedical Imaging Research, Tsinghua University, Beijing, China; d The Mountain-Whisper-Light Statistics, Seattle, WA, USA; e Center for Child Health, Behavior and Development, Seattle Children׳s Research Institute, Seattle, WA, USA; f Department of Mechanical Engineering, University of Washington, Seattle, WA, USA; g Presage Biosciences, Inc., Seattle, WA, USA

**Keywords:** AIM-HIGH, Atherothrombosis Intervention in Metabolic Syndrome with Low HDL/High Triglyceride and Impact on Global Health Outcomes, DCE-MRI, dynamic contrast-enhanced magnetic resonance imaging, *K*^*trans*^, transfer constant, *V*_*p*_, fractional plasma volume, Atherosclerosis, Plaque, Magnetic resonance imaging, Carotid artery, Inflammation, Statin

## Abstract

This brief data article summarizes the clinical risk factors and laboratory data of a group of subjects recruited for the AIM-HIGH trial (Atherothrombosis Intervention in Metabolic Syndrome with Low HDL/High Triglycerides and Impact on Global Health Outcomes) and an associated magnetic resonance imaging (MRI) substudy. The sample is restricted to those on statin therapy at the time of enrollment and data are presented stratified by whether dynamic contrast enhanced MRI (DCE-MRI) markers of carotid plaque vascularity and inflammation were available or not. The data provided herein are directly related to the article “Longer Duration of Statin Therapy is Associated with Decreased Carotid Plaque Vascularity by Magnetic Resonance Imaging” [2].

**Specifications Table**

**Table:** 

Subject area	*Biology*
More specific subject area	*Cardiology*
Type of data	*Table*
How data was acquired	*Questionnaire, physical examination and fasting blood draw*
Data format	*Table of statistics*
Experimental factors	*Subjects were from the AIM-HIGH trial. All were 45 years or older with atherogenic dyslipidemia, clinically established cardiovascular disease and currently on statin therapy.*
Experimental features	*As part of an MRI substudy, some subjects underwent carotid DCE-MRI to assess vascularity and possible inflammation of carotid plaques. Clinical data was collected prospectively through case report forms and fasting blood draws.*
Data source location	*Seattle, WA, USA*
Data accessibility	*The data is presented within this article.*

**Value of the data**

• These data represent a detailed characterization of this clinically relevant cohort, which is drawn from a high-risk, secondary prevention population, already treated with statins; one key question regarding this type question is what other factors can be used to predict residual risk of secondary cardiovascular events.
• These data can also be used to identify potentially important differences between the carotid DCE-MRI subsample and the remainder of the cohort which could affect interpretation of results from DCE-MRI studies based on this cohort.
• Additionally, these data can be used to identify potential factors associated with obtaining usable or unusable DCE-MRI measurements; this information could be used to reduce exclusions in future studies.
• Lastly, these data can be used to identify possible subgroups for further analysis or collaboration; the given sample sizes of subgroups defined by demographics or risk factors are important inputs for power calculations needed to determine the feasibility of a substudy of this cohort.

## 1 Data

The data presented in this report consist of summaries of clinical atherosclerosis risk factors and laboratory measurements of the patient population from the AIM-HIGH (Atherothrombosis Intervention in Metabolic Syndrome with Low HDL/High Triglycerides and Impact on Global Health Outcomes) trial. Summaries are provided for subgroups based on inclusion in or exclusion from the DCE-MRI substudy [2].

## 2 Experimental design, materials and methods

### 2.1 Study participants

The data were collected from participants of the AIM-HIGH trial and MRI substudy. The AIM-HIGH trial inclusion and exclusion criteria have been published previously [1]. Briefly, the primary AIM-HIGH inclusion criteria included: (1) age 45 years or older, (2) documented stable coronary, cerebrovascular/carotid or peripheral arterial disease, and (3) “atherogenic dyslipidemia”, defined as HDL-cholesterol<40 mg/dL in men or <50 mg/dL in women, triglycerides 150–400 mg/dL; and LDL-cholesterol<180 mg/dL if not taking statin drugs. Only participants on statin therapy and with known prior duration of treatment were included in this data compilation.

MRI substudy specific inclusion criteria were: (1) eligible for main AIM-HIGH study, (2) medically able to undergo MRI procedure, (3) willing to provide informed consent for sub-study participation. Substudy specific exclusion criteria were: (1) history of pacemaker or metallic implants, (2) history of bilateral carotid endarterectomy, or (3) estimated glomerular filtration rate less than 60 mL/min/1.73 m^2^. This study was approved by the AIM-HIGH Executive Committee and a local institutional review board or research ethics committee at each participating clinical site. Separate signed informed consent was obtained from each participant in this substudy.

### 2.2 Clinical and laboratory data collection

Demographics (sex, age, race and ethnicity), clinical history (prior statin use, prior niacin use, history of diabetes) and other atherosclerosis risk factor data (tobacco use, metabolic syndrome, blood pressure, body mass index) were collected prospectively by a questionnaire and physical examination and were recorded using case report forms (CRFs). The duration of statin therapy prior to entering the trial was recorded using the categories <1 year, 1–5 years and >5 years. This categorization was determined by the AIM-HIGH trial executive committee prior to data analysis in this study. At the baseline examination to confirm eligibility for the trial, a fasting blood draw was performed. Lipids (triglycerides and total, LDL-, HDL-cholesterol) and lipoproteins (ApoB, ApoA1 and lipoprotein(a)) were measured from these samples.

### 2.3 DCE-MRI protocol

The carotid DCE-MRI protocol and image analysis procedures are described in detail elsewhere [2], [3]. Prior to image analysis, participants were excluded if image quality was insufficient to interpret the images or there was a DCE-MRI protocol violation which prevented proper estimation of the DCE-MRI parameters. In addition, cases were excluded during the image analysis if the mean carotid vessel wall thickness was found to be <1 mm due to low reliability of DCE-MRI estimates in this situation. While DCE-MRI measurement data are not presented here, more information regarding the protocol, image analysis and reproducibility has been reported [4].

### 2.4 Statistical methods

Categorical variables were summarized as percentage (count) and continuous variables were summarized as mean±standard deviation (SD). Clinical and laboratory values were compared between groups of subjects using Fisher’s exact test (categorical variables) and the Mann–Whitney test (continuous variables). Three groups (not mutually exclusive) were defined: (1) AIM-HIGH subjects with DCE-MRI measurements available, (2) subjects who underwent DCE-MRI but did not have usable measurements, and (3) AIM-HIGH subjects without usable DCE-MRI measurements available (excludes group 1 but contains group 2). Only subjects on statin therapy at enrollment with known prior duration were included. All statistical calculations were conducted using R (version 2.14.1; The R Foundation for Statistical Computing, Vienna, Austria). Throughout, two-tailed tests were used with statistical significance defined as *p*<0.05.

## 3 Data tables

Of the 3414 participants randomized in the AIM-HIGH trial, 3101 (91%) were on statin therapy at entry with known prior duration. Of these, 206 underwent DCE-MRI. After excluding 52 for image quality issues or protocol violations and 56 for wall thickness below the minimum threshold of 1 mm, there were 98 subjects available for analysis in the DCE-MRI substudy. Table 1 summarizes the demographics, clinical status and laboratory data of the 98 DCE-MRI participants and the remainder of the cohort. Table 2 compares clinical and laboratory data between the 98 DCE-MRI participants with usable measurements and the 108 DCE-MRI participants without usable measurements (excluded for image quality, protocol violations or wall thickness below the minimum, as described above).

## Funding sources

This AIM-HIGH sub-study was supported by National Heart, Lung, and Blood Institute (NHLBI) grants R01-HL089504 (to Dr. O’Brien) and R01-HL088214 (to Dr. Zhao). No commercial entity provided any direct support for this substudy, nor did any commercial entity have any role in substudy oversight, design, or data analysis and interpretation.

The parent trial, AIM-HIGH, was supported by NHLBI Grants U01-HL-081616 and U01-HL-081649, as well as by an unrestricted grant from Abbott Laboratories (now AbbVie), Chicago, IL, as well as drug donations from Abbott Laboratories and from Merck.

## Figures and Tables

**Table 1:** Baseline summary of DCE-MRI, clinical and laboratory data in the DCE-MRI substudy sample and those in the remaining AIM-HIGH cohort who were on statin therapy at enrollment.

**Variable**	**In DCE-MRI substudy**		***P*-value**
**Yes (*N*=98)**	**No (*N*=2903)**
Sex – no. (%)			>0.99
Male	84 (86%)	2464 (85%)	
Female	14 (14%)	439 (15%)	
Age – years	62±9	64±9	0.072
Race^a^ – no. (%)			0.69
White	90 (92%)	2690 (93%)	
Non-white/other	8 (8%)	212 (7%)	
Ethnicity^a^ – no. (%)			0.79
Hispanic or Latino	4 (4%)	111 (4%)	
Not Hispanic or Latino	93 (96%)	2792 (96%)	
Tobacco use^a^ – no. (%)			0.48
Current	21 (21%)	558 (19%)	
Former (quit>1 year ago)	48 (49%)	1580 (55%)	
Never used	29 (30%)	742 (26%)	
Duration of statin therapy – no. (%)			0.052
<1 year	21 (21%)	371 (13%)	
1–5 years	39 (40%)	1217 (42%)	
>5 years	38 (39%)	1315 (45%)	
Prior use of niacin – no. (%)	15 (15%)	568 (20%)	0.36
Presence of metabolic syndrome^a^ – no. (%)	79 (81%)	2334 (81%)	>0.99
History of diabetes – no. (%)	22 (22%)	979 (34%)	**0.022**
Body mass index^a^ – kg/m^2^	30±4.1	31±5.3	**0.005**
Systolic blood pressure^a^ – mmHg	129±18	128±16	0.61
Diastolic blood pressure^a^ – mmHg	75±10	74±10	0.85
Total cholesterol – mg/dl	139±23	144±25	0.31
LDL cholesterol – mg/dl	71±18	72±21	0.95
Non-HDL cholesterol – mg/dl	105±21	109±24	0.32
Triglycerides – mg/dl	169±62	181±66	**0.037**
Lipoprotein(a)^a^ – mg/dl	84±90	77±89	0.55
ApoB^a^ – mg/dl	82±19	82±19	0.98
HDL cholesterol – mg/dl	34±6.1	35±5.6	0.62
ApoA-I^a^ – mg/dl	119±16	124±16	**0.039**
Total:HDL ratio	4.2±0.8	4.2±0.9	0.60
ApoB:ApoA-I ratio^a^	0.7±0.2	0.7±0.2	0.17

Unless otherwise specified, values are no. (%) or means±SD.

a Individuals missing values excluded for comparisons involving that variable, including: race (*n*=1), ethnicity (*n*=1), tobacco use (*n*=23), metabolic syndrome (*n*=9), body mass index (*n*=5), systolic blood pressure (*n*=2), diastolic blood pressure (*n*=3), lipoprotein(a) (*n*=48), apoB (*n*=54), apoA-I (*n*=54) and apoB:apoA-I ratio (*n*=54).

**Table 2:** Comparison of baseline variables between those with DCE-MRI measurements and those with DCE-MRI but who were excluded due to image quality, protocol violations or wall thickness<1 mm.

**Variable**	**DCE-MRI subjects**^a^		***P*-value**
**Included (*N*=98)**	**Excluded (*N*=108)**
Sex – no. (%)			0.46
Male	84 (86%)	88 (81%)	
Female	14 (14%)	20 (19%)	
Age – years	62±9	61±8	0.30
Race^b^ – no. (%)			**0.026**
White	90 (92%)	86 (80%)	
Non-white/other	8 (8%)	21 (20%)	
Ethnicity^b^ – no. (%)			0.38
Hispanic or Latino	4 (4%)	8 (7%)	
Not Hispanic or Latino	93 (96%)	100 (93%)	
Tobacco use – no. (%)			0.48
Current	21 (21%)	17 (16%)	
Former (quit>1 year ago)	48 (49%)	61 (56%)	
Never used	29 (30%)	30 (28%)	
Duration of statin therapy – no. (%)			0.76
<1 year	21 (21%)	28 (26%)	
1–5 years	39 (40%)	40 (37%)	
>5 years	38 (39%)	40 (37%)	
Prior use of niacin – no. (%)	15 (15%)	22 (20%)	0.37
Presence of metabolic syndrome – no. (%)	79 (81%)	87 (81%)	>0.99
History of diabetes – no. (%)	22 (22%)	32 (30%)	0.27
Body mass index^b^ – kg / m^2^	29.7±4.1	30.9±4.8	0.064
Systolic blood pressure^b^ – mmHg	129±18	128±17	0.87
Diastolic blood pressure^b^ – mmHg	75±10	75±9	0.72
Total cholesterol – mg/dl	139±23	141±28	0.92
LDL cholesterol – mg/dl	71±18	73±25	0.91
Non-HDL cholesterol – mg/dl	105±21	107±28	0.88
Triglycerides – mg/dl	169±62	171±63	0.78
Lipoprotein(a)^b^ – mg/dl	83.6±89.8	73.5±92.0	0.41
ApoB^b^ – mg/dl	82.2±19.0	82.5±21.4	0.97
HDL cholesterol – mg/dl	34.3±6.1	34.9±5.2	0.62
ApoA-I^b^ – mg/dl	118.9±16.4	123.9±16.7	0.10
Total:HDL ratio	4.2±0.8	4.1±1.1	0.67
ApoB:ApoA-I ratio^b^	0.7±0.2	0.7±0.2	0.29

Unless otherwise specified, values are no. (%) or means±SD.

a Only includes those already on statins at enrollment with known prior duration.

b Individuals missing values excluded for comparisons involving that variable, including: race (*n*=1), ethnicity (*n*=1), body mass index (*n*=1), systolic blood pressure (*n*=1), diastolic blood pressure (*n*=1), lipoprotein(a) (*n*=2), apoB (*n*=2), apoA-I (*n*=2) and apoB: apoA-I ratio (*n*=2).
