# Supplementary material for: Summary of clinical and laboratory data of study subjects with and without DCE-MRI plaque measurements in the AIM-HIGH clinical trial
Source: Data Brief. 2016 Jan 2;6:476–81. doi: 10.1016/j.dib.2015.12.030 (PMC4773570; doi:10.1016/j.dib.2015.12.030)
Supplement: Supplementary file 1 — Supplementary material [file mmc1.pdf]

CONFLICT OF INTEREST DECLARATION

This AIM-HIGH sub-study was supported by National Heart, Lung, and Blood Institute (NHLBI) grants R01-HL089504 (to Dr. O'Brien) and R01-HL088214 (to Dr. Zhao). No commercial entity provided any direct support for this sub-study, nor did any commercial entity have any role in sub-study oversight, design, or data analysis and interpretation.

The parent trial, AIM-HIGH, was supported by NHLBI grants U01-HL-081616 and U01-HL-081649, as well as by an unrestricted grant from Abbott Laboratories (now AbbVie), Chicago, IL, as well as drug donations from Abbott Laboratories and from Merck.

Outside of the present study, Dr. Yuan reports grants from the NIH and Philips Medical. He also serves as a Member of Radiology Advisory Network, Philips. Mr. Hippe reports grants from GE Healthcare. No other authors have any other potential conflicts of interest to disclose.

We confirm that the manuscript has been read and approved by all named authors and that there are no other persons who satisfied the criteria for authorship but are not listed. We further confirm that the order of authors listed in the manuscript has been approved by all of us.

We confirm that we have given due consideration to the protection of intellectual property associated with this work and that there are no impediments to publication, including the timing of publication, with respect to intellectual property. In so doing we confirm that we have followed the regulations of our institutions concerning intellectual property.

We further confirm that any aspect of the work covered in this manuscript that has involved human patients has been conducted with the ethical approval of all relevant bodies and that such approvals are acknowledged within the manuscript.

We understand that the Corresponding Author is the sole contact for the Editorial process (including Editorial Manager and direct communications with the office). He is responsible for communicating with the other authors about progress, submissions of revisions and final approval of proofs. We confirm that we have provided a current, correct email address which is accessible by the Corresponding Author and which has been configured to accept email from cardiac@uw.edu.

Signed by all authors as follows:

Name: Kevin D. O'Brien **Signature:** **Date:** 6·29·15

Name: Daniel S. Hippe **Signature:** **Date:** 5/11/15

Name: Huijun Chen **Signature:** **Date:**

Name: Moni B. Neradilek **Signature:** **Date:**

Name: Jeffrey L. Probstfield **Signature:** **Date:** 6-17-15

Name: Suzanne Peck **Signature:** **Date:**

Name: Daniel A. Isquith **Signature:** **Date:**

Name: Gador Canton **Signature:** **Date:** 05/11/15

Name: Chun Yuan **Signature:** **Date:** 5/14/15

Name: Nayak L. Polissar **Signature:** **Date:**

Name: Xue-Qiao Zhao **Signature:** **Date:**

Name: William S. Kerwin **Signature:** **Date:** 5/19/15

Name: Kevin D. O'Brien **Signature:** **Date:**

Name: Daniel S. Hippe **Signature:** [illegible] **Date:** 5/11/15

Name: Huijun Chen **Signature:** Huijun Chen **Date:** 5/12/2015

Name: Moni B. Neradilek **Signature:** **Date:**

Name: Jeffrey L. Probstfield **Signature:** **Date:**

Name: Suzanne Peck **Signature:** **Date:**

Name: Daniel A. Isquith **Signature:** **Date:**

Name: Gador Canton **Signature:** Gador Canton **Date:** 05/11/15

Name: Chun Yuan **Signature:** **Date:**

Name: Nayak L. Polissar **Signature:** **Date:**

Name: Xue-Qiao Zhao **Signature:** **Date:**

Name: William S. Kerwin **Signature:** **Date:**

**Name:** Kevin D. O'Brien	**Signature:**	**Date:**
**Name:** Daniel S. Hippe	**Signature:** [illegible]	**Date:** 5/11/15
**Name:** Huijun Chen	**Signature:**	**Date:**
**Name:** Moni B. Neradilek	**Signature:** [illegible]	**Date:** 5/12/15
**Name:** Jeffrey L. Probstfield	**Signature:**	**Date:**
**Name:** Suzanne Peck	**Signature:**	**Date:**
**Name:** Daniel A. Isquith	**Signature:** [illegible]	**Date:** 5/12/2015
**Name:** Gador Canton	**Signature:** Gador Canton	**Date:** 05/11/15
**Name:** Chun Yuan	**Signature:**	**Date:**
**Name:** Nayak L. Polissar	**Signature:**	**Date:**
**Name:** Xue-Qiao Zhao	**Signature:** Xue-Qiao Zhao	**Date:** 5/12/15
**Name:** William S. Kerwin	**Signature:**	**Date:**

**Name:** Kevin D. O'Brien	**Signature:**	**Date:**
**Name:** Daniel S. Hippe	**Signature:**	**Date:** 5/11/15
**Name:** Huijun Chen	**Signature:**	**Date:**
**Name:** Moni B. Neradilek	**Signature:**	**Date:**
**Name:** Jeffrey L. Probstfield	**Signature:**	**Date:**
**Name:** Suzanne Peck	**Signature:**	**Date:**
**Name:** Daniel A. Isquith	**Signature:**	**Date:** 5/12/2015
**Name:** Gador Canton	**Signature:**	**Date:** 05/11/15
**Name:** Chun Yuan	**Signature:**	**Date:**
**Name:** Nayak L. Polissar	**Signature:**	**Date:**
**Name:** Xue-Qiao Zhao	**Signature:** Xue-Qiao Zhao	**Date:** 5/12/15
**Name:** William S. Kerwin	**Signature:**	**Date:**

**Name:** Kevin D. O'Brien	**Signature:**	**Date:**
**Name:** Daniel S. Hippe	**Signature:**	**Date:** 5/11/15
**Name:** Huijun Chen	**Signature:**	**Date:**
**Name:** Moni B. Neradilek	**Signature:**	**Date:**
**Name:** Jeffrey L. Probstfield	**Signature:**	**Date:**
**Name:** Suzanne Peck	**Signature:**	**Date:** 5/13/2015
**Name:** Daniel A. Isquith	**Signature:**	**Date:**
**Name:** Gador Canton	**Signature:**	**Date:** 05/11/15
**Name:** Chun Yuan	**Signature:**	**Date:**
**Name:** Nayak L. Polissar	**Signature:**	**Date:**
**Name:** Xue-Qiao Zhao	**Signature:**	**Date:**
**Name:** William S. Kerwin	**Signature:**	**Date:**

**Name:** Kevin D. O'Brien	**Signature:**	**Date:**
**Name:** Daniel S. Hippe	**Signature:** [illegible]	**Date:** 5/11/15
**Name:** Huijun Chen	**Signature:**	**Date:**
**Name:** Moni B. Neradilek	**Signature:**	**Date:**
**Name:** Jeffrey L. Probstfield	**Signature:**	**Date:**
**Name:** Suzanne Peck	**Signature:**	**Date:**
**Name:** Daniel A. Isquith	**Signature:**	**Date:**
**Name:** Gador Canton	**Signature:** Gador Canton	**Date:** 05/11/15
**Name:** Chun Yuan	**Signature:**	**Date:**
**Name:** Nayak L. Polissar	**Signature:** Nayak L. Polissar	**Date:** 5/12/15
**Name:** Xue-Qiao Zhao	**Signature:**	**Date:**
**Name:** William S. Kerwin	**Signature:**	**Date:**
